# Coupling Computational Fluid Dynamics Simulations and Statistical Moments for Designing Healthy Indoor Spaces

**DOI:** 10.3390/ijerph16050800

**Published:** 2019-03-05

**Authors:** Shamia Hoque, Firoza B. Omar

**Affiliations:** Department of Civil and Environmental Engineering, University of South Carolina, 300 Main Street, Columbia, SC 29208, USA; firoza.b.omar@gmail.com

**Keywords:** respiration, dead zones, dispersion, dosage, breathing zone, office

## Abstract

Cross-contamination between occupants in an indoor space may occur due to transfer of infectious aerosols. Computational fluid dynamics (CFD) provides detailed insight into particle transport in indoor spaces. However, such simulations are site-specific. This study couples CFD with statistical moments and establishes a framework that transitions site-specific results to generating guidelines for designing “healthy” indoor spaces. Eighteen cases were simulated, and three parameters were assessed: inlet/outlet location, air changes per hour, and the presence/absence of desks. Aerosol release due to a simulated “sneeze” in a two-dimensional ventilated space was applied as a test case. Mean, standard deviation, and skewness of the velocity profiles and particle locations gave an overall picture of the spread and movement of the air flow in the domain. A parameter or configuration did not dominate the values, confirming the significance of considering the combined influence of multiple parameters for determining localized air-flow characteristics. Particle clustering occurred more when the inlet was positioned above the outlet. The particle dispersion pattern could be classified into two time zones: “near time”, <60 s, and “far time”, >120 s. Based on dosage, the 18 cases were classified into three groups ranging from worst case scenario to best case scenario.

## 1. Introduction

Computational fluid dynamics (CFD) has been used as a powerful simulation platform for air flow, thermal distribution, and contaminant and particle transport in the indoor environment for the past 20 years and more. Different CFD models for a range of geometries and ventilation patterns were generated and validated [1,2,3,4,5,6]. The simulations gave detailed insight into the influence of building parameters and indoor air quality (IAQ). However, the knowledge gained from CFD simulations is yet to benefit or influence decision-making or guideline development regarding exposure and infections for occupants of an interior space. 

The literature published on CFD and indoor air, from 1994 to 2018, dropped from ~1400 to less than 150 and 20 when refined by the words “exposure” and “infection”. Spenglar and Chen [7] in 2000 in the Annual Review of Energy and Environment showcased the potential of CFD to become the design tool for the future, especially to meet the requirement of healthy indoor environments. IAQ must ensure the “health” and “well-being” of the residents as declared by World Health Organization (WHO) in 2000 [8]. ASHRAE standards (62.1 and 62.2) evolved over the years to provide occupants with health, comfort, and productivity [9]. In recent years, indoor environments and, hence, IAQ took on new dimensions with factors such as designing for energy efficiency and sustainability. Guidelines focused on thermal, acoustic, and visual comforts, as well as sick building syndrome [10]. The impact of IAQ on the occupants is multi-fold and is a result of the interactions between the parameters influencing IAQ and the factors impacting health and well-being of residents [11]. There is strong and sufficient evidence to establish relationships between ventilation, air-flow pattern, and the spread of infectious diseases in buildings [12]. One route of transmission is via aerosols, defined as “person-to-person transmission of pathogens through the air by means of inhalation of infectious particles”. 

Studies show that ventilation rates of 25 L/s per person have the potential to reduce sick building syndrome and sick leave, while low ventilation rates at schools can have a negative impact on school absence and respiratory illness [3]. The efficiency of a ventilation system is tied to the task it is performing [4,5]. Office design has some impact on performance, e.g., women in open-floor plans reported higher long-term sick leave spells [6]. However, increasing ventilation rates can also lead to a negative impact with increased outdoor pollutants coming indoors. Personalized designs, such as introducing chair fans in conjunction with displacement ventilation were also studied to reduce exposure of occupants to pollutants, i.e., particles in the breathing zone [7]. Further investigations into assessing parameters influencing exposure risk between occupants showed that a person-to-person distance of less than 1.1 m in an office increases chances of infection [11]. 

We spend 90% of our time indoors, and buildings are responsible for the consumption of 20% to 40% of energy in the developed world [13]. To ensure acceptable IAQ levels, without increasing energy costs, identifying and assessing the influence of indoor space parameters such as location of desks and windows relative to engineering controls such as vent locations is vital. Different ventilation patterns were tested to identify the ideal location for a printer so as to reduce particle concentration in the breathing zone [14], and ventilation strategies, particle concentration, and removal efficiency were shown to be dependent on source location [15]. CFD simulations can extract detailed information on the influence of the micro space parameters and IAQ, but the information provided is based on a specific location and its application for general guidelines is limited. 

In this investigation, a series of simulations are conducted varying different parameters, and the worst and best exposure scenarios are determined through dosage estimation. Idealized two-dimensional large eddy simulations (2D LES) are conducted to understand and extract the “interferences” of the various parameters. The 2D LES approach was applied to understand downstream effects for high-rise buildings [16] and gave accurate information regarding the mechanisms governing vortex shedding around bluff bodies [17]. The study explores coupling CFD simulations with statistical approaches. It proposes a framework that can lead to applying three-dimensional (3D) CFD results in generating guidelines and standards for “healthy” indoor spaces, reducing the transfer of infectious aerosols between occupants. The study assesses the potential of statistical moments to translate CFD data on velocity profiles and particle transport into information relating to indoor conditions and possibility of exposure. 

## 2. Methodology

### 2.1. Numerical Model Development

**Figure 1:**
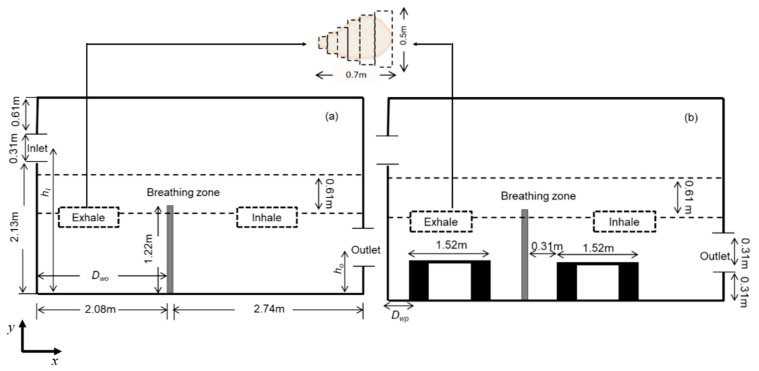
Problem scenario: (**a**) partition only; (**b**) partition and desks. Dimensions are in meters.

**Computational domain:** The geometry of the computational domain for a representative case is shown in Figure 1a,b. The source of particles in the space is due to respiratory release represented as a “sneeze”. The dimensions of the 2D space are 4.88 × 3.05 m^2^, representing an office room shared by two occupants. The partition wall height is 1.22 m and is located 2 m from the inlet side. The inlet and outlet positions and the presence or absence of desks are dependent on the specific case as listed in Table 1. A total of 18 cases are simulated. The parameters varied to change the scenario for the cases are *h_I_* and *h_O_*, which are the height of the inlet and outlet from the floor of the domain, respectively; *D_wp_*, which is the distance from the wall to the partition in the exhale zone; and *D_wo_*, which is the distance from the wall or the side where the inlet is located to the first obstruction encountered from that wall. The “breathing zone” is in the range of 1 to 2 m from the floor. This zone is further separated into the exhalation and inhalation zones. In the “exhalation zone”, the residing occupant sneezes at a height of 1.07 m from the floor, representing a person sitting at a desk, and the “inhalation zone” is where the second office occupant may inhale the exhaled aerosols. It is assumed that the presence of any aerosol in the breathing zone has the potential of causing infection. The sneeze particles are released in an area measuring 0.7 m in length and 0.5 m in height. The area is divided into six segments where 150,000 aerosols are injected randomly with a velocity range of 6–22 m/s with a particle size of 7 μm, assuming a sneeze volume of 1 L [11,18]. To simulate the high momentum at the point of release and the subsequent loss of energy, it is assumed that the particles released close to the source have the maximum velocity, and, as the distance increases, the particle velocity decreases. 

**Mathematical formulation:** The Eulerian–Lagrangian framework was applied to simulate the transport of particles in the indoor space. Details of the development were given in Reference [19], and the numerical approach was validated against different geometries [20]. The flow dynamics of the room were captured via large eddy simulation (LES) [21,22]. LES provides an instantaneous velocity field required to calculate the particle trajectories [22]. Equations (1) and (2) solve for mass and momentum conservation, respectively, where $u_{i}$ is the velocity component in *x*, *y*, *z* directions, and $\tau_{ij}$ is the sub-grid scale stress term which was resolved by applying the Smagorinsky model, $\tau_{ij}-\tau_{kk}\delta_{ij}/3=2\nu_{t}{\bar{S}}_{ij}$, where ${\bar{S}}_{ij}=\left(\partial {\bar{u}}_{i}/\partial x_{j}+\partial {\bar{u}}_{j}/\partial x_{j}\right)/2$; finally, the eddy viscosity *ν_t_* is obtained from $\nu_{t}=C_{s}\Delta^{2}\left|\bar{S}\right|{\bar{S}}_{ij}$, where ∆ is the grid size, *C_s_* is the Smagorinsky constant, and $\left|\bar{S}\right|={\left(2{\bar{S}}_{ij}{\bar{S}}_{ij}\right)}^{1/2}$ [23].

(1) $$\partial {\bar{u}}_{i}/\partial x_{i}=0;$$

(2) $$\frac{\partial {\bar{u}}_{i}}{\partial t}+\frac{\partial }{\partial x_{j}}\left({\bar{u}}_{i}{\bar{u}}_{j}\right)=-\frac{1}{\rho }\frac{\partial \bar{p}}{\partial x_{i}}-\frac{\partial \tau_{ij}}{\partial x_{j}}+\nu_{t}\frac{\partial^{2}{\bar{u}}_{i}}{\partial x_{j}\partial x_{j}}.$$

Particle trajectories were determined applying the force balance equation $\sum F_{i}=m\left(du_{P}/dt\right)=F_{D}+F_{G}$, where *u_p_* is the particle velocity of mass *m*, at location *x_i_*, *F_G_* is the net gravitational force, $F_{G}=\left(1/6\right)\pi d_{p}{}^{3}\left(\rho_{p}-\rho \right)g$, and *F_D_* is the drag force, $F_{D}=\left(1/8C\right)\pi d_{p}{}^{2}\rho C_{D}\left|V_{R}\right|V_{R}$. *C* is the Cunningham correction factor, *C_D_* is the drag coefficient, *V_R_* is the relative velocity, *d_p_* is the particle diameter, *ρ_p_* is particle density, and *ρ* is fluid density. 

**Boundary conditions:** The boundary condition of the walls of the room represents no-slip conditions. Uniform velocity profiles are specified at the inlets. The outlets are pressure boundaries with Dirichlet conditions and Neumann conditions applied for all other dependent variables. Particle trajectory calculations terminated when they exited the room. The simulation assumed that the aerosols were spherical and neutral, and no energy loss occurred during its interaction with surfaces. This was assumed to simulate the worst-case scenario, i.e., all expirated aerosols remain in the ambient atmosphere for the longest duration. No evaporation occurs, i.e., the particle size remains constant. One-way coupling was applied, and resuspension was not considered. 

**Solver settings:** The equations were discretized based on finite volume techniques having second-order accuracy for time and spatial derivatives. The numerical simulations were conducted using CFD-ACE+ [24]. A blended second-order upwind scheme was applied to resolve the convective diffusive terms, and the temporal terms were solved via the implicit Euler scheme. The convergence criterion was set at 10^−7^. For each case, the flow field was first generated for 240 s, based on air changes per hour and the dimensions of the geometry; this ensured the domain was filled with air before particle release via a “sneeze”. The simulation was terminated 300 s after the sneeze. 

**Grid independence:** A structured computational grid was applied with finer grid resolution at the boundaries, the furniture, and partitions. The grid independence was tested using three resolutions for the case in Figure 1. In LES, to obtain grid independence, often the grid has to be refined until it nearly reaches a DNS (direct numerical simulation)-level resolution and, as a result, loses the LES fundamentals. Hence, the grid convergence index (GCI) approach [25,26] was applied to assess the uncertainty associated with each step of grid resolution for predicting the number of particles remaining in the room, applying $GCI=F_{s}\left[\left(f_{coarse}-f_{fine}\right)/\left(1-r^{P}\right)\right]$, where $f=\left(N/N_{o}\right)$, *F_s_* = 3 (safety factor), *r* = grid ratio, and *p* = 2 (formal order of accuracy). The GCI index was 8% when the grids were refined from 25,000 to 50,000, and <2% when the grids went from 50,000 to 80,000, indicating a grid resolution of 50,000 results in an error margin of <2% compared to 80,000. In the current study, the grid resolution of 50,000 was chosen for all the simulations.

### 2.2. Design and Analysis Plan

**Design plan:** To assess the impact of (1) relative inlet/outlet location, *h_I_/h_o_*, (2) air changes per hour (ACH), and (3) presence and absence of desks, *D_wo_/D_wp_*, the design plan was executed as follows:(1) when the inlet and outlet of the domain were located at the top, the ratio *h_I_/h_o_* = 1; when the inlet and outlet were located at opposite walls with the inlet positioned higher than the outlet, *h_I_/h_o_* > 1; and when the inlet was located on the opposite wall but positioned lower than the outlet, then *h_I_/h_o_* < 1, (2)the air changes per hour were set at three levels: 3, 5, and 7 and (3) for the scenarios with desks, the ratio *D_wo_/D_wp_* < 1, and in the cases without desks, *D_wo_/D_wp_* < 1. Table 1 summarizes the dimensional configuration of the 18 cases with the corresponding schematic. 

**Analysis approach:** Spatial and temporal data on velocity and particles were extracted from all cases. The effects of the parameters, *h_I_/h_o_*, ACH, and *D_wo_/D_wp_*, on the air flow pattern and on the temporal and spatial particle distribution in the total domain and in the breathing zone were assessed. Statistical moments were applied to describe and distinguish these scenarios and the dosage was calculated. Parameter configurations significantly influencing air flow and particle distribution were determined. The analysis was done with the MATLAB’s statistical package [27].

## 3. Results and Analysis

### 3.1. Influence of Parameters on Air-Flow Pattern

**Figure 2:**
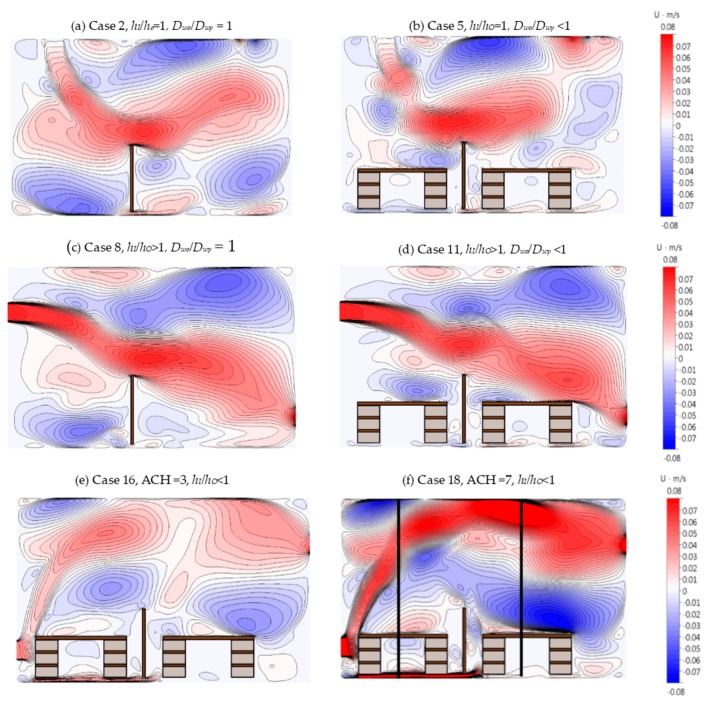
Velocity contour plots at air changes per hour (ACH) = 5 for (**a**) case 2, (**b**) case 5, (**c**) case 8, and (**d**) case 11; velocity contour plot at ACH = 3 for (**e**) case 16, and at ACH = 7 for (**f**) case 18.

The velocity contour plots in Figure 2 show the resulting air-flow pattern for cases 2, 5, 8, 11, 16, and 18. Cases 2, 5, 8, and 11 were at ACH = 5. In Figure 2a, where both inlet and outlet were in the ceiling (case 2), the circulations with higher velocities were in the upper regions of the room and at the corners. For case 5, Figure 2b, with all other configurations the same as case 2, the presence of desks did not appear to change the air-flow pattern significantly. In Figure 2c,d, cases 8 and 11 appear similar, even though desks were present for the latter and absent for the former. Figure 2e,f show the velocity contour plots for ACH = 3 and 7 when *h_I_/h_o_* < 1 in the presence of desks. The presence of the desks in front of the inlet resulted in a sharp upturn of the air flow at the inlet into the domain, resulting in a different air-flow pattern in comparison to when *h_I_/h_o_* = 1 or *h_I_/h_o_* > 1. The plots confirm the combined influence that indoor parameters have on the air flow in the domain, but it is difficult to distinguish which parameter or configuration is better or worse for the occupants’ well-being.

The contour plots do, however, confirm that increasing ACH resulted in fewer locations in the domain where the velocity magnitudes were <0.01 m/s. The regions where such low velocities occurred remained the same for all configurations, as seen in Figure 2. To assess the impact of the parameters on the regions with low velocities, the number of nodes with velocities less than 0.005 m/s was counted and normalized against the total number of nodes in the breathing and non-breathing zones. These were designated as dead zones. 

**Figure 3:**
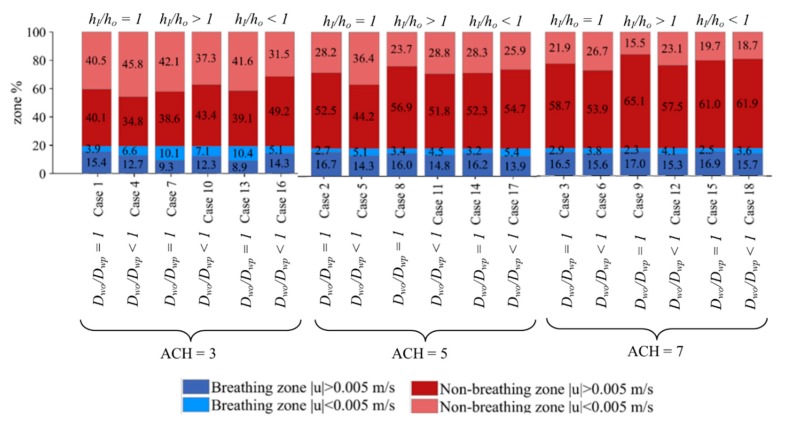
Percentage of dead zones in breathing and non-breathing parts of the domain.

The bar charts in Figure 3 compare the percentage of dead zones for the 18 cases. The breathing zones are in blue shades and non-breathing zones are in red shades. The figure shows that increasing ACH, i.e., cases 1, 2, and 3 or cases 4, 5, and 6, resulted in a decreasing percentage of dead zones in the whole domain and in the breathing zone. The percentage of dead zones in the domain nearly halved when air changes were increased from 3 to 5, though the decrease when ACH was increased from 5 to 7 was not as consistent.

Comparing cases without desks (*D_wo_/D_wp_* = 1) and with desks (*D_wo_/D_wp_* < 1), the percentage of dead zones in both regions increased when the inlet and outlet were located at the top, for ACH = 3 (cases 1 and 4). When *h_I_/h_o_* > 1 (cases 7 and 10) and *h_I_/h_o_* < 1 (cases 13 and 16), the dead zone percentage decreased with the inclusion of desks for the whole domain. At ACH = 5, dead zone percentage increased for all regions when *h_I_/h_o_* = 1 (cases 2 and 5) and *h_I_/h_o_* > 1 (cases 8 and 11), and it increased for the breathing zone only when *h_I_/h_o_* < 1 (cases 14 and 17). The dead zone percentage decreased for the non-breathing zones for all cases, with cases 14 and 17 as the only exceptions, when desks were included. At ACH = 7, the exception also occurred when *h_I_/h_o_* < 1 (cases 15 and 18) for the non-breathing zone as well, with a slight increase in the percentage of dead zones when desks were included. Overall, it appears that, when the inlet and outlet location satisfies *h_I_/h_o_* < 1, the trend differs from the other configurations. This can be due to the location of the desks in the domain relative to the inlet position.

### 3.2. Line Plots and Statistical Moments for Interpreting Air Flow across All Cases

**Figure 4:**
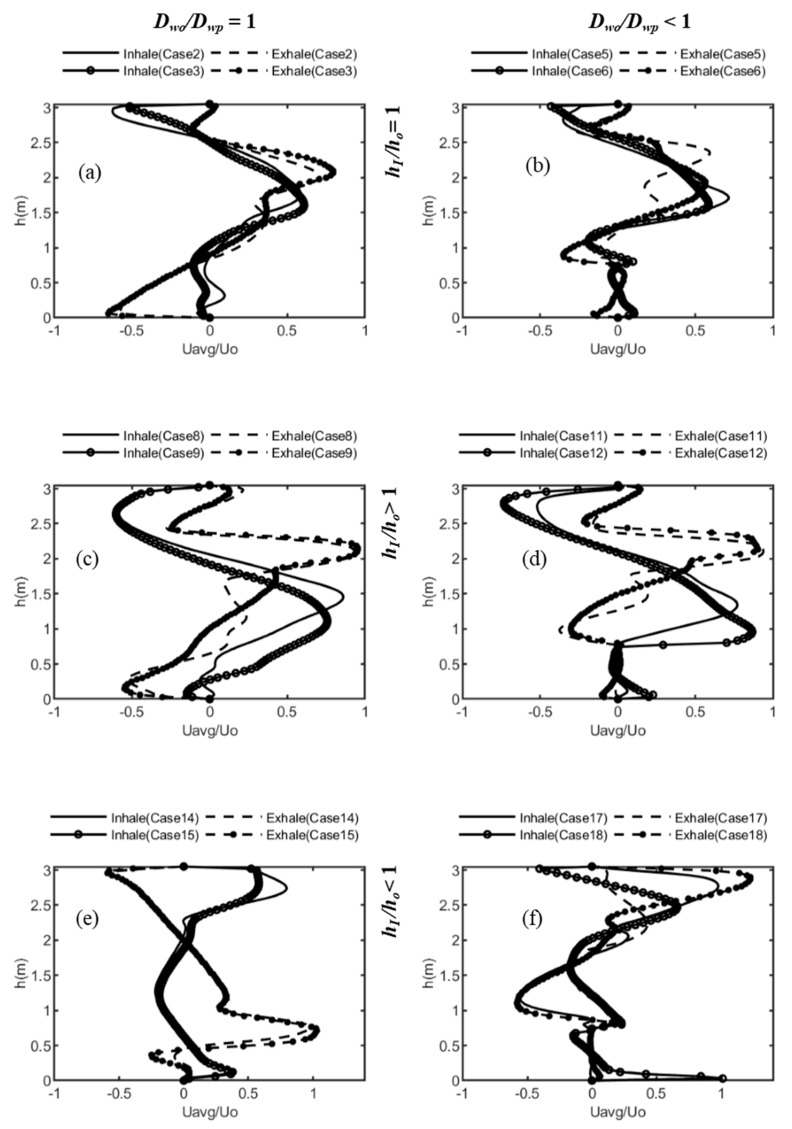
Average velocity line plots; solid and dotted lines are the normalized velocities across the height of the room at exhale (*x* = 1.1 m) and inhale (*x* = 3.05 m) locations, respectively; lines without markers are for ACH = 5 and those with markers are for ACH = 7; (**a**) cases 2 and 3, (**b**) cases 5 and 6 (**c**) cases 8 and 9, (**d**) cases 11 and 12, (**e**) cases 14 and 15, and (**f**) cases 17 and 18.

Figure 4 shows line plots of the normalized velocity for cases 2, 3; 5, 6; 8, 9; 11, 12; 14, 15; and 17, 18 at *x* = 1.1 m and 3.05 m in the exhale and inhale zones (locations shown in Figure 2d using line probes). Figure 4a compares the velocity profiles for cases 2 and 3 where ACH increases from 5 to 7. The air-flow inlet and outlet were located at the top, i.e., *h_I_/h_o_* = 1, and no desks were present, *D_wo_/D_wp_* = 1. Increasing the ACH caused a vortex or recirculation zone to form near the domain for ACH = 7; otherwise, the line plots were similar for both locations. Figure 4b shows cases 5 and 6 with desks, which resulted in velocities near zero at desk heights. Figure 4c,d show line plots for cases 8, 9; and 11, 12, with the inlet located above the outlet, *h_I_/h_o_* > 1. Maximum velocity values occurred at the inlet height for the exhale side, with the lines nearly overlaying. For the inhale side, the plots appeared to “smooth” out as inlet effects diminished (*h_I_/h_o_* < 1 for Figure 4e,f). The peaks were reversed for the exhale and inhale zones in Figure 4e. The presence of desks resulted in sharp peaks at the lower end of the domain (Figure 4f). Overall, the position of the inlet/outlet on the flow pattern appeared to have a lesser effect when *h_I_/h_o_* > 1, and desks appeared to have a lesser effect when *h_I_/h_o_* = 1. Figure 4 shows the influence of the different configurations on the air-flow pattern, and it highlights the difficulty in comparing the effects of the multiple configurations. 

To better interpret the results across all 18 cases, statistical moments were applied next. The mean, standard deviation, and the skewness were calculated for the average velocities across the room height at *x* = 1.1 m and 3.05 m. The means increased from ~0.004 m/s to ~0.007 m/s to ~0.010 m/s as the ACH increased from 3 to 7, and they were very nearly the same value for both sides of the partition. Table 2 lists the values for standard deviation and skewness for both exhale and inhale zones, and the change going from one zone to the other. The standard deviation increased with increasing ACH for each zone. Comparing exhale and inhale sides, an impact of the configuration can be seen. The standard deviation decreased from the exhale to the inhale region when *h_I/_h_o_* = 1 and *D_wo_/D_wp_* = 1. There was a slight increase when desks were present and ACH = 5 and 7. This indicates that the air flow does not gain momentum for this configuration, suggesting the possibility that contaminant dispersion is influenced mainly by the conditions at the inlet side of the domain. With desks present, the air flow was interrupted and, at higher ACH, some momentum was carried forward, resulting in a rise in the standard deviation. A decreasing trend from the exhalation zone to the inhalation zone can be seen for *h_I/_h_o_* < 1 in the absence of any desks. In the presence of desks, however, the standard deviation increased for all cases moving from the exhale to the inhale side for *h_I/_h_o_* > 1.

Skewness gives the direction of the total mass of air. A negative skew indicates that the mass of the air flow is toward *x* = 0, and a positive skew shows that the mass of air is flowing toward the room end, i.e., where the outlet is located. In the exhale zone, when the inlet and outlet were located at the top, the mass of air flow was toward the inlet, i.e., skewness was negative for cases 1 to 4. In all other cases, for the exhale zone, the air flow was directed toward the outlet. Cases 1, 2, and 3 had no desks. With no desks to break the incoming air stream from the inlet located in the ceiling, the air mass had more space to move in either or both directions. This can also be seen for case 4, where, at ACH = 3, even though desks were present, the air dispersed before the air stream hit the desks. 

For the inhale zone, cases 2, 9, 12, and 16 had negative skew values. All these cases had unique configurations, and skewness gives insight into the impact of the different configurations. Case 2 is the only case for the group with *h_I/_h_o_* = 1 where the flow of the air mass was toward the exhale zone from the inhale zone (ACH = 5 and no desks were present). For case 9, the group of cases 7, 8, and 9 had the same parameter values (*h_I/_h_o_* > 1, *D_wo_/D_wp_* = 1), except for increasing ACH from 3 to 5 to 7, respectively. Cases 10, 11, and 12 were also the same (*h_I/_h_o_* > 1, *D_wo_/D_wp_* < 1) except for ACH. One group was without desks and the other was with desks. At ACH = 7, the flow direction was opposite for cases 9 and 12. Case 16 with ACH = 3, on the other hand, also had a negative skew value. For case 16, *h_I/_h_o_* < 1 and desks were present. Skewness transitioned from negative to positive and vice versa for the cases 1, 3, 4, 9, 12, and 16. For cases 1, 3, and 4, where the common factor was *h_I/_h_o_* = 1, the transition was negative to positive. For cases 9, 12 (*h_I/_h_o_* > 1), and case 16 (*h_I/_h_o_* < 1), the transition was positive to negative, i.e., the mass of air moved toward the exhale zone, away from the inlet end, and then reversed direction. 

### 3.3. Statistical Moments Describing the Spatial Distribution of Particles

**Figure 5:**
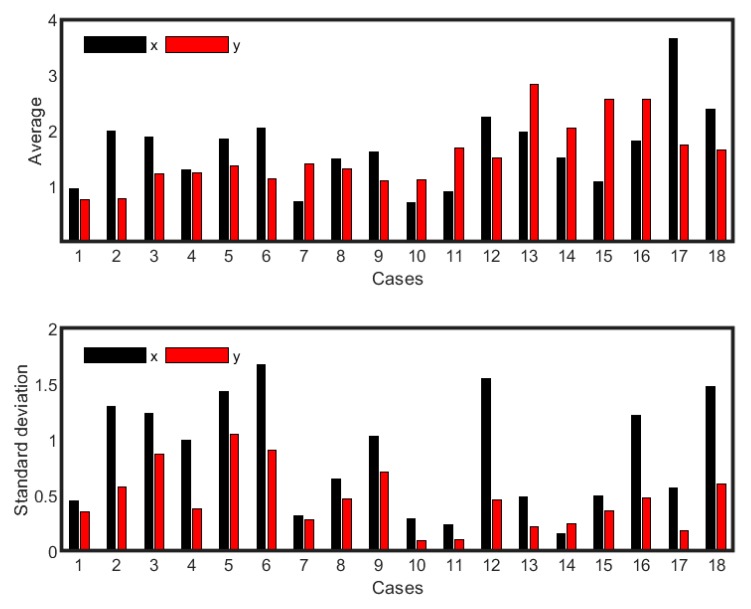
(**a**) Average (m) of *x* and *y* locations for the 18 cases; (**b**) standard deviation (m) for *x* and *y* locations for the 18 cases at the end of simulation.

To obtain an overall picture of the spatial distribution of the particles, the average and standard moments of the location of the particles for *x* and *y* coordinates at the end of the simulation were determined. Figure 5 shows the average and standard deviation (*x* and *y* coordinates) for the 18 cases. The values differed for all cases, which confirms that every configuration of ventilation pattern, ACH, and desks resulted in a unique spatial distribution of the particles. A higher value of the mean or average *x* and *y* indicated that particles were located toward the inhalation zone or that more particles were located near the ceiling, respectively. Standard deviation quantifies the clustering of the particles in the domain around the average. For example, particles in case 2 (ACH = 5, *h_I/_h_o_* = 1, *D_wo_/D_wp_* = 1) were, on average, within 2 m of the entrance, occupying the region right below the breathing zone, but dispersed more in the horizontal direction, staying within a meter of the domain’s floor. For case 17 (ACH = 5, *h_I/_h_o_* < 1, *D_wo_/D_wp_* < 1), on the other hand, particles moved to the inhalation zone and were in the breathing region, but clustered in that location.

Increasing ACH from 3 to 5 generally pushed the particles toward the inhale zone, for example, in cases 1, 2; and cases 7, 8; except for cases 13, 14 where *h_I_/h_o_* < 1 and *D_wo_/D_wp_* = 1, and for cases 17, 18 where *h_I_/h_o_* < 1 and *D_wo_/D_wp_* < 1. There was no clear trend when ACH increased from 5 to 7. Most particles remained in the exhale zone for case 7. For case 17, most particles appeared to be in the inhale zone. For cases 13 to 16, the particles were above the breathing zone, whereas, for all the remaining scenarios, the particles were within the height of the breathing zone. Cases 13 to 16 had the common configuration of *h_I_/h_o_* < 1. Once ACH increased from 3 to 5 and 7 for cases 17 and 18, the particles were at the height within the breathing zone.

**Figure 6:**
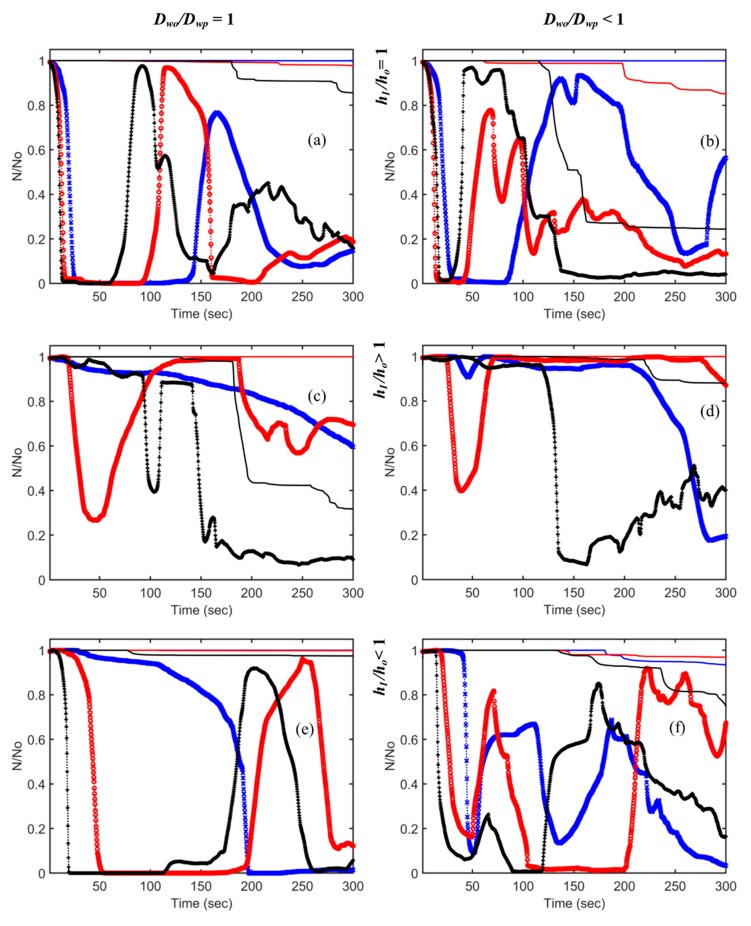
Temporal change of particle number in the domain and in the breathing zone (solid lines—whole domain; lines with markers—breathing zone; blue, red, and black represent ACH = 3, 5, and 7, respectively): (**a**) cases 1, 2, and 3; (**b**) cases 4, 5, and 6; (**c**) cases 7, 8, and 9; (**d**) cases 10, 11, and 12; (**e**) cases 13, 14, and 15; (**f**) cases 16, 17, and 18.

Scanning through the standard deviation values (Figure 6b), it could be concluded that no specific parameter appears to dominate the particle dispersion for both *x* and *y* directions. Increasing ACH from 3 to 5 resulted in an increase in the standard deviation value for *x* when the inlet and outlet were located at the top, i.e., *h_I/_h_o_* = 1 (cases 1, 2; cases 4, 5). For *h_I/_h_o_* > 1 (cases 7, 8; cases 10, 11) and *h_I/_h_o_* < 1 (cases 13, 14; cases 16, 17), the increase was not consistent. When ACH = 7, higher spread occurred for case 12 (ACH = 7, *h_I/_h_o_* > 1, *D_wo_/D_wp_* < 1) and case 18 (ACH = 7, *h_I/_h_o_* < 1, *D_wo_/D_wp_* < 1). However, particles in case 16 (ACH = 3, *h_I/_h_o_* < 1, *D_wo_/D_wp_* < 1) also had a standard deviation value nearly the same as cases 12 and 18, even though the ACH was 3. The least particle dispersion occurred for the cases 7, 10, 11, and 14 for the *x* value. The values for *y* location followed the same trend as *x* and were always less than *x*, except for case 14 (ACH = 5, *h_I/_h_o_* < 1, *D_wo_/D_wp_* = 1), where dispersion of the particles was slightly more in the vertical direction than in the horizontal direction.

The standard deviation values for air flow in Table 2 were compared to the trends in Figure 5b. Focusing on cases with relatively more clustering for both *x* and *y* values (i.e., cases 7, 10, 11, 14, and 17), it can be seen that smaller magnitudes of standard deviation for air flow were seen for cases 7 (~0.019 both sides) and 10 (~0.013 both sides) when ACH = 3 and *h_I/_h_o_* > 1 in the absence and presence of desks, respectively, compared to the other cases. However, for cases 11, 14, and 17, the standard deviation for air flow was within the magnitude of the other cases (~0.021 to ~0.029) even though the standard deviation values for particles indicated clustering. In these three cases, the inlet and outlet were at the opposite end (case 11, *h_I/_h_o_* > 1; case 14, *h_I/_h_o_* < 1; and case 17, *h_I/_h_o_* < 1) and ACH was either 5 (case 11, 17) or 7 (case 14). Desks were present for cases 11 and 17 but absent for case 14. 

### 3.4. Temporal Trend of the Particles

The temporal evolution of the particle number in the breathing zone and in the whole domain for all cases is shown in Figure 6. The plots show the unique impact of each configuration of the 18 simulations. The effect of increasing ACH from 3 to 7 is shown in every plot. The first column in the figure is for the cases with the presence of the partition only, *D_wo_/D_wp_* = 1, and the second column is for the cases with both partition and desks, *D_wo_/D_wp_* < 1. The first row is for *h_I_/h_o_* = 1, the second row is for *h_I_/h_o_* > 1, and the third row is for *h_I_/h_o_* < 1. Lines in the plot represent particle number evolution in the whole domain, and lines with markers represent evolution in the breathing zone only. 

Assessing the influence of increasing air changes, at ACH = 7, a larger number of particles left the domain compared to at ACH 3 and 5. However, the least removal also occurred when *h_I_/h_o_* < 1 and *D_wo_/D_wp_* = 1 for ACH = 7 (case 15). There is no clear interpretation as to the impact of the presence or absence of desks or the inlet/outlet locations. When the inlet and outlet were located at the top, in the presence of desks, more particles exited the room for ACH = 5 and 7. There was little or no change in the trend for ACH = 3. When *h_I_/h_o_* < 1 for ACH = 5 and 7, the presence of desks was observed to have the same effect; however, overall, a smaller number of particles were removed when compared to *h_I_/h_o_* = 1. For *h_I_/h_o_* > 1, the presence of desks resulted in the entrapment of higher particles in the whole domain, and the number leaving the domain was small. Particles cycled in and out of the breathing zone with the air flow. The peak of the cycles was dependent on the total remaining particles in the domain. Hence, more particles returned to the breathing zone in the following cycle if particles remained in the room. Cases 13, 14, and 15 (Figure 6e) illustrate this clearly. For ACH = 3, it appears that particles in the breathing zone left the room; however, for ACH = 5 and 7, the pattern indicates a return earlier for ACH = 7 than that for ACH = 5. Hence, for ACH = 3, the particles return to the breathing zone after a longer time period. The peak of the cyclic behavior coincides with the trend line for the change in the total number of particles in the domain.

**Figure 7:**
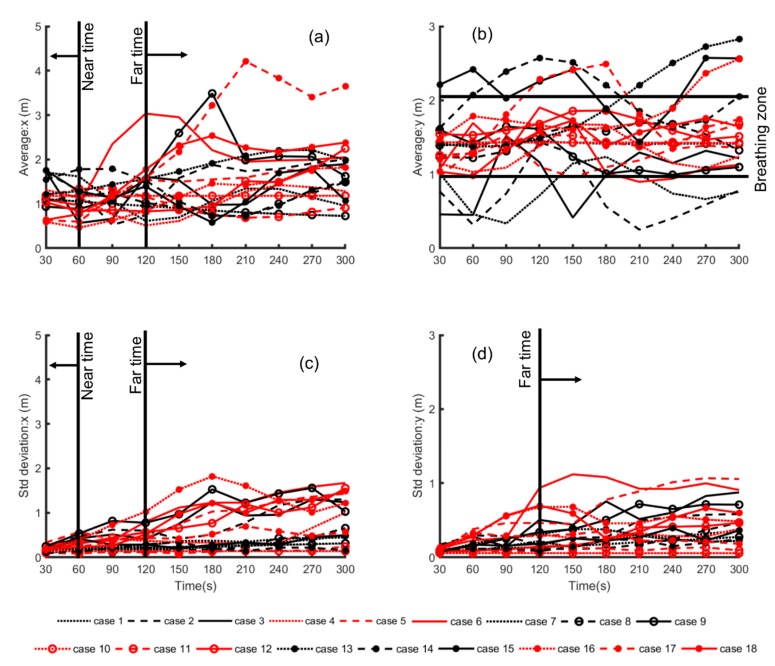
(**a**) Average *x*; (**b**) average *y*; (**c**) standard devaiton of *x*; and (**d**) standard deviation of *y* for particle locations. Black = *D_wo_/D_wp_* = 1, red = *D_wo_/D_wp_* < 1; dotted lines: ACH = 3, dashed lines: ACH = 5, and solid lines: ACH = 7; no marker: *h_I_/h_o_* = 1, “o”: *h_I_/h_o_ >1*, and “*”: *h_I_/h_o_ < 1*.

To assess the temporal trend for all cases, the average and standard deviation change over time for the *x* and *y* locations of the particles in the whole domain and in the breathing zone were plotted (Figure 7). The dispersion trend of the particles could be classified under “near time”, <60 s after release, and “far time”, >120 s after release, along the horizontal direction of the room (Figure 7a,c). For the first 60 s, the average location of the particles was on the inlet side, around the exhale zone, before dispersing afterward. After that, the effects of the room configuration appeared to take over with the average value of *x* location, i.e., the spatial distance from the inlet side or the exhale region, increasing. The value of *x* was mainly higher for higher ACH and for the configuration *h_I_/h_o_* > 1, while it was lower for ACH = 3. Figure 7c is the corresponding temporal change of the standard deviation of the spatial *x* locations. The standard deviation is a representation of the particle clustering trend. Within the first minute of release, the standard deviation was ~0.5 or less for all cases. After 120 s, the particles started dispersing for some scenarios, and, for others, the particles remained clustered for the entire particle tracking duration. 

Figure 7b shows a distinct difference in the average location of the particles along the room height. Particles released in the configuration where the inlet/outlet was located at the top appeared to congregate in the lower portion of the space (below 1.5 m) for all ACH and in the presence and absence of desks. Case 7 was the only exception, where the particles constantly stayed at the same average height. For all cases, the particles congregated in and around the breathing zone. Figure 7d shows the corresponding standard deviation change of the particle positions for *y*. The standard deviation increased beyond 120 s.

### 3.5. Dosage

The total number of particles inhaled over time was calculated using Equation (3), where *D_t_* is the dosage or the number of particles inhaled over a specific time period *t*, $N_{p}^{t}$ is the number of particles (maximum, minimum, or mean) in the breathing zone for time *t*, *f* is the fraction of particles that will enter the respiratory tracts based on a particle size of 0.05, and $f_{R}^{t}$ accounts for breathing over the time period *t*, assuming a representative adult population and that the amount of particles that can be breathed in *t* time is $N_{p}^{t}$ [28].

(3) $$D_{t}=N_{p}^{t}\times f\times f_{R}^{t}.$$

**Figure 8:**
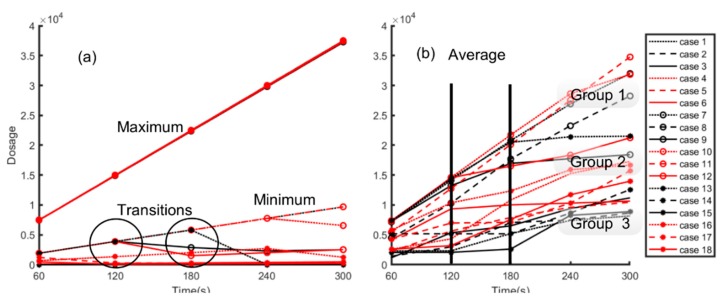
(**a**) Maximum and minimum, and (**b**) average number of particles that an adult is exposed to after a “sneeze” over 300 s or a five-minute period.

Figure 8a shows the results for the maximum and minimum dosage for the 18 cases, and Figure 8b shows the change in average dosage over 300 s. The maximum inhaled dosage increased exponentially and overlapped for all cases, with the maximum value at 4 × 10^4^. For the minimum value, it varied with the highest being near 1 × 10^4^ for cases in which *h_I_/h_o_* > 1 and desks were present with the exception of when ACH = 7 (case 12). The minimum hovered near zero and decreased for the other configurations of *h_I_/h_o_*. There appeared to be a change in the behavior after 120 s and then after 180 s. 

Figure 8b captures the average number of particles exposed to over the time period. The plot is broken down into three groups. Group 1 refers to the cases where there was an exponential rise. The average dosage in group 1 was also closer to the maximum line in Figure 8a. Group 2 refers to cases which had an exponential rise until 120 s or 180 s and then leveled off. The dosage amounts also fell between the maximum and minimum boundaries. Group 3 covers the remaining cases, which fell near the minimum band of Figure 8a. Some cases of this group also started with an exponential rise, but this rise was less steep than that in group 1. Others transitioned into an exponential rise after 120 s or 180 s. Table 3 classifies the groups and the related cases with their specific configurations. In group 1, *h_I/_h_o_* > 1 for all cases and ACH = 7 was absent. Hence, the “worst-case scenario” occurred when the inlet was located above the outlet for lower air changes. At higher air changes, the effects of the location were minimized. The six cases in group 2 were equally divided with two cases each for the ratio *h_I/_h_o_*, i.e., when ACH = 7, *h_I/_h_o_* > 1, and when ACH = 3, *h_I/_h_o_* < 1, further illustrating the inter-dependent relationship between the relative location of inlet/outlet and air changes per hour. In group 3, which can be classified as the “best-case scenario”, there were four cases for the inlet and outlet located at the top, and the remaining four cases for the inlet located below the outlet. 

The influence of the presence or absence of desks was neutral, comparing the number of cases in group 1. In group 2, there were more cases with desks than without desks. Transitions at different times occurred in the cases in group 2, indicating that desks influenced the outcomes. Group 3 justified having no desks, as cases where no desks were present dominated the group, indicating the inhalation dosage of particles increased for the occupants when desks were present. 

## 4. Conclusions

This study established a framework of extracting information from CFD simulations and coupling it with statistical moments for identifying guidelines for minimizing exposure to occupants. Eighteen cases were simulated applying a 2D LES Eulerian–Lagrangian framework. The impact of the inlet/outlet location (*h_I/_h_o_)*, air changes per hour (ACH), and presence/absence of desks (*D_wo_/D_wp_*) was assessed. If inlet/outlet were both located on the ceiling, then *h_I/_h_o_* = 1; if the inlet was located above the outlet, then *h_I/_h_o_* > 1; and if the inlet was located below the outlet, then it was assigned as *h_I/_h_o_* < 1. ACH was assigned three values (3, 5, or 7), and desk presence was denoted by *D_wo_/D_wp_* < 1 and *D_wo_/D_wp_* = 1. The effects on the air-flow pattern and particle trajectories were unique for the different configurations. To interpret the information and translate it to guidelines, the average velocity and particle number in the room was assessed using the first three moments: mean, standard deviation, and skewness. 

Mean, standard deviation, and skewness of the velocity profiles gave an overall picture of the spread and movement of the air flow in the domain. Inlet/outlet locations influenced the change in the standard deviation of the velocities at the exhale and inhale zones, with the air flow “spreading” into the domain for *h_I_/h_o_* < 1. Skewness captured the transition of mass of air-flow direction going from negative (toward exhale zone) to positive (toward inhale zone). A single parameter or configuration did not dominate, confirming the significance of considering the combined influence of multiple parameters for determining localized air-flow characteristics. The spatial distribution of the particles and the temporal variation of their number was also tracked. The average and standard deviation of the *x* and *y* locations of the particles at the end of the simulation were calculated. For *h_I_/h_o_* < 1, more particles stayed above the breathing zone compared to other scenarios. There was a clear trend of particles being located near the outlet for ACH increasing from 3 to 5, while, from 5 to 7, the same conclusion could not be drawn. However, more particles left the room for ACH = 7 from the whole domain compared to other values of ACH. More particle dispersion occurred in the *x* direction when the inlet and outlet were located at the top, with more clustering occurring for *h_I_/h_o_* > 1, with the influence of ACH dominating when ACH = 7. 

The temporal change of the average and standard deviation values of *x* and *y* showed that the particle dispersion pattern could be classified into two time zones: “near time”, <60 s, and “far time”, >120 s. While the maximum dosage inhaled was the same for all scenarios, the minimum exposure trend changed after 120 s, with the exposure either decreasing or continuing to rise, with another change occurring in the trend at 180 s for a few cases. Based on the average dosage intake, the 18 cases were separated into three groups. The relative dosage that the occupants were exposed to was dependent on multiple variables. The combination of the different variables resulted in “groups” of configurations ranging from worst-case to best-case scenario. The cases in group 3 (best-case scenario) all had decreasing standard-deviation trends for velocity profiles (except for case 1 which had a slight increase). All air changes were present in this group. The position of the inlet above the outlet was absent in this group. Group 2 had cases where skewness transitioned, and the dosage trend flattened after an initial exponential rise. The dead zone percentages in the breathing regions for the cases in groups 1 to 3 were compared. No specific trend was noted. For the worst-case scenario, the inlet was always above the outlet, and ACH = 7 was absent. These results can vary based on the physics introduced and by changing the geometry to a 3D domain. In a 3D domain, mixing will be enhanced and, hence, the spatial and temporal distribution, and the associated dosage will differ. However, by identifying the minimum, average, and maximum dosage trends, the best- and worst-case scenarios can be categorized. The framework can be applied to identify design approaches that may limit exposure. Additionally, the results can guide the selection of cases that might have to be simulated in a 3D domain to extract additional information and insight. 

The 2D LES approach gave a clear picture of the influence of the multiple variables and different configurations on the air-flow pattern and particle transport. The simulation results coupled with the inferences from statistical moments gave a map toward utilizing the information from more detailed simulations. However, the flow obtained was not a perfect representation of a real-life flow field in a 3D room, and the effects resulting from changing the room configuration in each case were, therefore, enhanced to a certain level. To capture the 3D physics governing the flow and particle transport, the framework established in the study can be extended to a series of 3D simulations. The steps outlined in the current study will have to be expanded to account for the influence of the third dimension. The results obtained will change with the introduction of physico-chemical mechanisms governing particle fate and transport as required, such as the evaporation of the sneeze droplets in a space maintained at a specific temperature and humidity. 

## Figures and Tables

**Table 1:** Dimensional configuration of the 18 cases simulated. ACH—air changes per hour.

Cases	*h_I_/h_O_*	ACH	*D_wo_/D_wp_*	Schematic
**1**	1	3	1	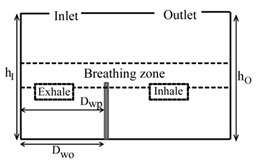
**2**	1	5	1	
**3**	1	7	1	
**4**	1	3	<1	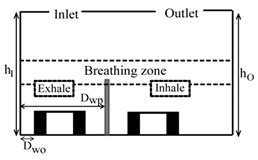
**5**	1	5	<1	
**6**	1	7	<1	
**7**	>1	3	1	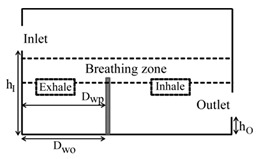
**8**	>1	5	1	
**9**	>1	7	1	
**10**	>1	3	<1	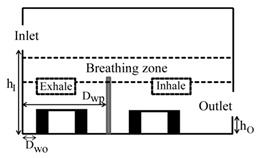
**11**	>1	5	<1	
**12**	>1	7	<1	
**13**	<1	3	1	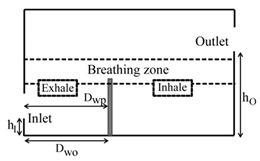
**14**	<1	5	1	
**15**	<1	7	1	
**16**	<1	3	<1	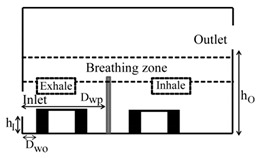
**17**	<1	5	<1	
**18**	<1	7	<1	

**Table 2:** Standard deviation and skewness to assess the changes from exhale to inhale zones.

Cases	Standard Deviation			Skewness			Parameters
	Exhale	Inhale	Change	Exhale	Inhale	Change	
**1**	0.0154	0.0157	SI	−0.3578	0.5745	− to +	ACH = 3, *h_I/_h_o_* = 1, *D_wo_/D_wp_* = 1
**2**	0.0244	0.0211	D	−0.2670	−0.5117	−	ACH = 5, *h_I/_h_o_* = 1, *D_wo_/D_wp_* = 1
**3**	0.0369	0.0271	D	−0.0778	0.2837	− to +	ACH = 7, *h_I/_h_o_* = 1, *D_wo_/D_wp_* = 1
**4**	0.0130	0.0097	D	−0.1462	0.0773	− to +	ACH = 3, *h_I/_h_o_* = 1, *D_wo_/D_wp_* < 1
**5**	0.0145	0.0205	D	0.6520	0.5867	+	ACH = 5, *h_I/_h_o_* = 1, *D_wo_/D_wp_* < 1
**6**	0.0240	0.0258	I	0.3090	0.2823	+	ACH = 7, *h_I/_h_o_* = 1, *D_wo_/D_wp_* < 1
**7**	0.0125	0.0194	I	1.8934	0.1022	+	ACH = 3, *h_I/_h_o_* > 1, *D_wo_/D_wp_* = 1
**8**	0.0250	0.0306	I	0.5296	0.0666	+	ACH = 5, *h_I/_h_o_* > 1, *D_wo_/D_wp_* = 1
**9**	0.0372	0.0444	I	0.5432	−0.0696	+ to −	ACH = 7, *h_I/_h_o_* > 1, *D_wo_/D_wp_* = 1
**10**	0.0130	0.0139	SI	1.6068	0.2702	+	ACH = 3, *h_I/_h_o_* > 1, *D_wo_/D_wp_* < 1
**11**	0.0219	0.0261	I	1.3355	0.1180	+	ACH = 5, *h_I/_h_o_* > 1, *D_wo_/D_wp_* < 1
**12**	0.0314	0.0449	I	1.1623	−0.1107	+ to −	ACH = 7, *h_I/_h_o_* > 1, *D_wo_/D_wp_* < 1
**13**	0.0148	0.0098	D	0.6818	1.4505	+	ACH = 3, *h_I/_h_o_* < 1, *D_wo_/D_wp_* = 1
**14**	0.0246	0.0202	D	0.5851	1.1315	+	ACH = 5, *h_I/_h_o_* < 1, *D_wo_/D_wp_* = 1
**15**	0.0375	0.0139	D	0.7027	0.8071	+	ACH = 7, *h_I/_h_o_* < 1, *D_wo_/D_wp_* = 1
**16**	0.0164	0.0261	I	0.6594	−0.3745	+ to −	ACH = 3, *h_I/_h_o_* < 1, *D_wo_/D_wp_* < 1
**17**	0.0144	0.0292	D	1.0527	0.4880	+	ACH = 5, *h_I/_h_o_* < 1, *D_wo_/D_wp_* < 1
**18**	0.0452	0.0259	D	0.9088	1.0448	+	ACH = 7, *h_I/_h_o_* < 1, *D_wo_/D_wp_* < 1

I = increase; D = decrease; SI = slight increase.

**Table 3:** Details of the configurations of the cases in each group.

Group 1		Group 2		Group 3	
Case	Configuration	Case	Configuration	Case	Configuration
7	ACH = 3, *h_I/_h_o_* > 1, *D_wo_/D_wp_* = 1	4	ACH = 3, *h_I/_h_o_* = 1, *D_wo_/D_wp_* <1	1	ACH = 3, *h_I/_h_o_* = 1, *D_wo_/D_wp_* = 1
8	ACH = 5, *h_I/_h_o_* > 1, *D_wo_/D_wp_* = 1	6	ACH = 7, *h_I/_h_o_* = 1, *D_wo_/D_wp_* <1	2	ACH = 5, *h_I/_h_o_* = 1, *D_wo_/D_wp_* = 1
10	ACH = 3, *h_I/_h_o_* > 1, *D_wo_/D_wp_ <1*	9	ACH = 7, *h_I/_h_o_* > 1, *D_wo_/D_wp_* = 1	3	ACH = 7, *h_I/_h_o_* = 1, *D_wo_/D_wp_* = 1
11	ACH = 5, *h_I/_h_o_* > 1, *D_wo_/D_wp_ <1*	12	ACH = 7, *h_I/_h_o_* > 1, *D_wo_/D_wp_* < 1	5	ACH = 5, *h_I/_h_o_* = 1, *D_wo_/D_wp_* < 1
		13	ACH = 3, *h_I/_h_o_* < 1, *D_wo_/D_wp_* = 1	14	ACH = 5, *h_I/_h_o_* < 1, *D_wo_/D_wp_* = 1
		16	ACH = 3, *h_I/_h_o_* < 1, *D_wo_/D_wp_* < 1	15	ACH = 7, *h_I/_h_o_* < 1, *D_wo_/D_wp_* = 1
				17	ACH = 5, *h_I/_h_o_* < 1, *D_wo_/D_wp_* < 1
				18	ACH = 7, *h_I/_h_o_* < 1, *D_wo_/D_wp_* < 1
